# Information needs of health care workers in developing countries: a literature review with a focus on Africa

**DOI:** 10.1186/1478-4491-7-30

**Published:** 2009-04-08

**Authors:** Neil Pakenham-Walsh, Frederick Bukachi

**Affiliations:** 1 Global Healthcare Information Network, Charlbury, Oxford, UK; 2 Department of Medical Physiology, University of Nairobi, Nairobi, Kenya

## Abstract

Health care workers in developing countries continue to lack access to basic, practical information to enable them to deliver safe, effective care. This paper provides the first phase of a broader literature review of the information and learning needs of health care providers in developing countries.

A Medline search revealed 1762 papers, of which 149 were identified as potentially relevant to the review. Thirty-five of these were found to be highly relevant. Eight of the 35 studies looked at information needs as perceived by health workers, patients and family/community members; 14 studies assessed the knowledge of health workers; and 8 looked at health care practice.

The studies suggest a gross lack of knowledge about the basics on how to diagnose and manage common diseases, going right across the health workforce and often associated with suboptimal, ineffective and dangerous health care practices. If this level of knowledge and practice is representative, as it appears to be, it indicates that modern medicine, even at a basic level, has largely failed the majority of the world's population. The information and learning needs of family caregivers and primary and district health workers have been ignored for too long. Improving the availability and use of relevant, reliable health care information has enormous potential to radically improve health care worldwide.

## Background

In developing countries, many health care workers have little or no access to basic, practical information [1-3]. Indeed, many have come to rely on observation, advice from colleagues and building experience empirically through their own treatment successes and failures. In the last decade, some important steps have been made towards meeting the information needs of the "upper" echelons of health professions (research and tertiary care), but remarkably little progress has been achieved in meeting the information needs of primary and district health care providers in the developing world [4-6]. This disparity is due to several factors, including unequal distribution of Internet connectivity, and also a failure of international "information for development" policies and initiatives, which have tended to focus on "innovative" Internet-based approaches for higher-level health professionals and researchers while ignoring, relatively speaking, other approaches that remain essential for the vast majority of primary and district health workers.

The "information poverty" of health workers in Africa is exacerbating what is clearly a public health emergency on a massive scale: increasing numbers of people are living in poverty, and many continue to be denied access to basic health care services; one in six children are not living to see their fifth birthday; and there is a massive increase in noncommunicable diseases in addition to the huge HIV/AIDS burden.

Health workers are at the centre of efforts to address this crisis. They are hampered by two main factors. First, there is a gross deficiency in the actual number of health workers in Africa, affecting all cadres. The "brain drain" depletes public sector health workers to critically low numbers, especially in rural areas. Second, there has been a remarkable lack of attention on understanding and addressing the needs of existing health workers themselves, and how they might be better supported to deliver safe, effective care. The information and training needs of health workers are fundamental. It is only by addressing these needs that we can hope to achieve the Millennium Development Goals.

## Purpose

The purpose of this review is to provide preliminary information about the information needs of health care providers in developing countries and to highlight ways to address the issue.

## Objectives

This review provides a preliminary glimpse of the journal literature on the information needs of health workers in developing countries (with a special focus on Africa), and ways in which information needs can be assessed.

The objective was to address the following questions:

• What is known about the health information needs of health workers in developing countries (with a special focus on Africa)?

• What processes and tools have been used to assess health information needs in developing countries? What can we learn from their limitations? How can we adapt and adopt successful tools?

## Methods

The Medline database was searched using the terms: (((((provider* OR practitioner* OR worker* OR nurs* OR doctor* OR physician* OR personnel OR assistant* OR dispenser* OR midwi* OR surgeon*))) AND ((need* OR require* OR access* OR want*))) AND ((information OR knowledge))) AND (((((("Developing Countries" [MeSH]))) OR (("Africa" [MeSH]))))). The search was limited to articles published in the last 10 years up to 31 December 2006.

The reviewers then identified articles that were (1) possibly or (2) highly likely to be relevant to the review, on the basis of title and abstract. The full text of articles identified as highly likely to be relevant were retrieved, and these articles provide the basis for this review.

The reviewers screened every article based on two questions: Did the article say anything about health care information and learning needs of health care providers? And did it say anything about methods of information needs assessment? Each article was then classified on the basis of: (1) whether information needs highlighted were reported by the health care providers (users) or by others; or simply inferred from lack of knowledge and practical skills; and (2) major areas of public health and clinical practice: maternal and child health (MCH), HIV/AIDS, sexually transmitted infections (STIs) and tuberculosis (TB), cardiovascular diseases (CVDs) and diabetes, general internal medicine, and others. Key points emerging from each article were collected and synthesized.

## Results

Of the 1762 (titles and abstracts) retrieved, 149 (8.5%) were identified as potentially relevant to the review. These 149 papers were classified as follows:

• MCH: 45 papers (30%)

• HIV/AIDS and STIs: 22 (15%)

• CVDs and diabetes: 15 (10%)

• general internal medicine: 11 (7.4%)

• malaria: 6 (4%)

• others (education and training, drugs and therapeutics, health information, mental health, oncology, ophthalmology, health policy, etc): 50 (34%).

Of the 149 papers identified as potentially relevant, 35 (23%) articles were considered highly likely to be relevant to the purpose of the review, on the basis of title and abstract (see Additional File 1).

### Key points emerging from individual papers

#### Studies that assessed information needs as perceived by health workers

Eight of the 35 studies looked at information needs as perceived by health workers, patients and family/community members. Seven of these were carried out in Africa (Gambia [7], Ghana [8], Kenya [9,10], South Africa [11], Uganda [12], one multicentre (East Africa) [13]) and one in Indonesia [14]. All eight studies used interviews and/or questionnaires. The studies covered the full range of possible study populations, including doctors (6), nurses (3), clinical officers (3), community health workers (1), patients (2) and families/community members (2). Areas of health were similarly varied: general (2), diabetes (2), epilepsy (2), care of the elderly (1), psychiatric disorders (1) and surgery (1).

The Ghana study [8] looked at all public-sector health workers (N = 7691) in three of the 10 regions of Ghana (Volta, Western and Brong Ahafo regions). Of all respondents (N = 6696), 97% perceived that continuing professional education (CPE) is necessary. Ninety percent recognized CPE as necessary to maintaining and improving professional knowledge and skills, while 7% saw it as necessary to gaining relief from routine.

A study from the Kenyatta National Hospital (Nairobi, Kenya) found that most (56%) of the 130 doctors working at Kenyatta expressed a need for further training to deal with psychiatric conditions in their patients [9]. Psychiatric disorders are extremely common, yet in Africa there is only one psychiatrist per million population (compared to 134 per million in the United States of America), so it is especially important that non-psychiatrically trained health workers, whether working in hospitals or the community, are able to deal effectively with common disorders.

A questionnaire survey of 37 East African surgeons found that they prefer electronic journals to textbooks [13]. (It should be noted, however, that the results of this study may well have been affected by reporting bias – see item 4 in "Methodology issues" below.) "Western" journals (defined as being published within Canada, the United Kingdom of the United States) were indicated as being the most useful by most of the respondents in their clinical (76% of respondents), teaching (73%), and research (68%) activities. Local journals, defined as those from the region where the physicians practise, were regarded as most useful by far fewer respondents for their clinical (22%), teaching (14%), and research (11%) activities. A total of 62% said that they would change their practice based on "Western" journal information, in contrast to only 11% who would change it based on information from local journals.

A multicentre survey (China, Egypt, Kenya, India, Thailand) of hospital doctors clearly showed that textbooks remain the most commonly used source of information about the management of common medical conditions; journals were less popular and computer searching was uncommon [15]. Local textbooks and journals were used more than those from North America and Europe, except in Kenya, where the opposite was true. By contrast, personnel in health centres have different information needs. One particular study in primary care health centres in rural Uganda found that the few books donated to the facilities were too technical, contained inappropriate content and were generally irrelevant to the local needs. As one doctor put it "...Information in some of the textbooks we have about paediatrics, public health, internal medicine and pathology is not very relevant to our current tropical health situation because they were written in the West ... the focus is not tropical medicine"[12].

#### Studies that assessed knowledge of health workers

Fourteen studies assessed the knowledge of health workers. Of these, seven were from Africa (Kenya 2, Gambia 2, Nigeria, Somalia, South Africa), two were multicentre (both including countries in Africa), and others were from Egypt (2), India, Pakistan and Saudi Arabia.

A study in Kenya identified inadequate national guidelines as a cause of insufficient knowledge and practice: "The knowledge of 50% on type of care [for umbilical cord] was incorrect by international standards, but was in keeping with Nursing Council of Kenya teaching." The authors "recommend that the local Nursing Council policy be updated, and that all primary HW receive education, or in-service re-education on appropriate cord care." Most of the respondents in this study (87%) indicated that they had acquired their knowledge about cord care during training, 9.4% from fellow health workers, and 3.1% from mothers [16].

A similar problem was highlighted in a South African study of doctors in public-sector primary health care centres [17]: "Many participants noted inconsistencies between the maximum OGLA [oral glucose lowering agent] doses in the South African Medicines Formulary and the doses mentioned in the guidelines. Consequently, there was confusion as to whether insulin should be introduced or the dose of OGLAs increased." The study showed a gap in knowledge and training on when and how to initiate insulin therapy for poorly controlled type 2 diabetes. Participating doctors stated that most of their undergraduate training had focused on hospital treatment of acute complications of diabetes rather than on practical diabetes management in a primary-care setting. Many did not know the benefits of insulin for poorly controlled type 2 diabetes. And contrary to information in their national guidelines, some of the doctors believed that insulin was not beneficial in obese patients, while others questioned its value in the presence of established complications. As one doctor reported: "For me insulin [was not an option]. It frightened me because I had no idea how to [determine] the dosage for the patient."

Similar lack of knowledge was found in a study from India: "Both patients and medical practitioners displayed a lack of comprehension of the need for constant disease monitoring and consistent approaches to tight glycaemic control" [18].

A questionnaire-based study from Saudi Arabia found that consultants, junior doctors and nurses in a large teaching hospital had poor knowledge of some of the basic techniques of blood pressure (BP) measurement. This included inconsistencies regarding the knowledge of cuff size, recording of diastolic BP, position of the arm and rate for deflating the cuff. Overall, 60% of the respondents had insufficient knowledge. Forty percent of the nurses tended to round up the BP to the next 10 mmHg [19].

In Nigeria, a cross-sectional study involving 56 randomly selected primary and district health care facilities and 1000 consecutive hypertensive patients found a considerable knowledge and awareness gap related to hypertension and its complications, both among patients and health care providers [20]. BP control rates were poor (28%, with systolic blood pressure (SBP) < 140 mmHg and diastolic blood pressure (DBP) < 90 mmHg) and drug prescription patterns were not evidence-based and cost-effective. The same study found that about half the patients were unaware of the beneficial effects of physical activity and avoidance of smoking. A substantial number were also unaware of the beneficial effects of a low-salt diet (25%) and a "heart-healthy" diet (30%).

A cross-sectional study in Somalia assessed the knowledge and practices of registered practitioners in management of TB [21]. Of 100 registered doctors, 53 were interviewed. Of these, 32 (64%) had treated TB patients during the previous year, but only one had notified the authorities. Only 33 (66%) knew the most important symptoms and only 32 (64%) were able to identify sputum-smear microscopy as the most important diagnostic test. Only four doctors prescribed the correct regimen and only seven advocated direct observation (DOTS) as recommended by the World Health Organization (WHO). Suboptimal knowledge was more common among doctors working in private practice.

A qualitative study in the Gambia [22] used semistructured interviews followed by group discussions to assess the knowledge, attitudes and practices of 22 trained Gambian traditional birth attendants (TBAs) in the prevention, recognition and management of postpartum haemorrhage (PPH). The TBAs had received six weeks' training. Although all the TBAs were illiterate, some information from training had been incorporated into their knowledge. For example, 20 of 22 TBAs were able to describe the correct sequence for management of the third stage of labour. However, the review highlighted the importance of relevance of content in training manuals.

There is now a general trend away from efforts to train TBAs, on the basis that some studies have had disappointing results; WHO and others are focusing more on scaling up skilled attendance and access to centres with trained midwives. But it remains unclear to what extent the reported failures of TBA training are due to inherent factors associated with, for example, the educability of TBAs, versus external factors such as the method of training or the appropriateness of training materials.

A study from Egypt [23] revealed that 90% of diabetic patients had poor knowledge of the disease, 80% had poor knowledge of complications and 96% had poor knowledge of how to control the disease. Older patients and those with a lower educational level were associated with the poorest knowledge. The authors suggest that more research is needed on the amount, type and channels of information that will have an impact on diabetics and their families. They specifically suggest that health workers need training to more effectively provide health education support for their diabetic patients. But a study from South Africa suggests this may be easier said than done: "You only have an average of six minutes per patient. By the time you've examined them and found out that they're diabetic and what their glucose level is, you cannot possibly educate somebody in three minutes [or less]" [17].

A review and informal observations by researchers at the University of Nairobi highlight the lack of skills of health personnel in diabetes and foot care, except in the very few referral centres. "Therefore even where there is a health facility, there may be no quality diabetes care/foot care on offer" [24].

A multicentre study [15] suggested that knowledge of appropriate investigations (but not therapeutics) for a hypothetical case of adult pneumonia was strongly associated with access to literature-based scientific evidence (access to medical library, use of local journals and local and developed-country textbooks). Only 54% of general hospital physicians identified the appropriate treatment.

A second multicentre study (21 hospitals in Bangladesh, Dominican Republic, Ethiopia, Indonesia, Philippines, Tanzania and Uganda) assessed knowledge of five important clinical problems: pneumonia, diarrhoea with dehydration, sepsis, severe malnutrition and hypoglycaemia. Three fourths of the doctors had inadequate knowledge in at least one area, compared with 91% of nurses and medical assistants. Knowledge was much better among doctors in teaching hospitals than doctors in district hospitals, but nurses and medical assistants had poor knowledge in both district and teaching hospitals [25-27].

#### Studies that assessed practice of health workers

Eight studies looked at the practice of health workers. Of these, four were in Africa (Burkina Faso, Kenya, South Africa, Tanzania), two were multicentre (both including Africa), one was from Egypt, and one from Pakistan.

In Pakistan, a cross-sectional survey of 1000 randomly selected (primary) general practitioners (GPs) from urban areas found that the majority (63%) relied on representatives from pharmaceutical companies for updates on information about antihypertensive medications [28]. Almost 80% used incorrect blood pressure cutoff values to diagnose hypertension in patients older than 60 years. Over 40% of GPs inappropriately used sedatives as their treatment of first choice; half of these GPs prescribed sedatives alone, while the other half prescribed sedatives in combination with an antihypertensive agent. The authors emphasize that this approach is inappropriate, not only because it contributes to undertreatment of hypertension but also because it increases the risk of drug dependency in subjects for whom use of a sedative may not be indicated.

Seventy percent of GPs "prescribed sublingual antihypertensive medications on an ad hoc basis for rapidly lowering BP in patients whose BP was considered markedly elevated at presentation to the clinic". The authors note that "use of sublingual antihypertensive medications should be avoided because of the risk of complications from unpredictable aggressive BP reduction."

In addition, half of the GPs administered intravenous medications to lower BP in their offices. Appropriate therapy for hypertension in the elderly was initiated by only 35% of GPs. By contrast, thiazide diuretics were rarely prescribed (4%). The authors of the study expressed special concern that 23% of GPs discontinued treatment once BP control was achieved.

A study in South Africa [29] assessed the management of severely malnourished children in two rural district hospitals. Data were collected through retrospective review of case records, with detailed studies of selected cases, structured observations of the paediatric wards and interviews with ward sisters and doctors. The combined case fatality rate for severe malnutrition was extremely high: 32%. Many children died during the first few days of treatment, likely causes being missed infection, hypoglycaemia and hypothermia due to lack of night feeds, cardiac failure due to overhydration from intravenous (IV) fluids and electrolyte imbalance due to use of diuretics.

The authors noted deviations from almost every key area of WHO guidelines: (1) overuse of IV fluids; (2) failure to give broad-spectrum antibiotics routinely; (3) inappropriate prescription of iron during the initial phase of treatment; and (4) inappropriate high-protein diet from the first day of admission. Nurses also mentioned that they were aware that things were not done properly, but felt they had no control over the situation. The authors report that a training programme has been jointly developed with staff, and has resulted in a marked reduction in case fatality.

A study in rural Burkina Faso [30] investigated the quality of drug prescriptions in nine health centres. Three hundred and thirteen outpatient consultations were studied by methods of guided observation. Additionally, interviews were held with the health care workers involved in the study. In 12% of cases the drug was heavily over- or underdosed (defined as less than 50% of the minimal dosage for antimalarials or antibiotics and more than 200% of the maximal dosage of any other drug with serious undesired effects). Errors in dosage occurred significantly more often in children under five years of age. Seven out of 21 pregnant women received drugs contraindicated in pregnancy. And two thirds of patients received no information on how long the drug had to be taken.

Surprisingly, "the professional training of the health workers was not found to play a significant role in prescribing habits." The same study also highlighted the importance of design of health care information materials: "When asked what could be improved in it, they stated that it is sometimes difficult to find the right page, that too often referral to the next level is recommended, that signs are not put in relation to the disease and that some common diseases are missing (such as hepatitis and skin fungus). Additionally more illustrations were requested."

A study in Tanzania [31] found that: "Approximately 87% of drugs were prescribed according to the essential drug list of Tanzania. This was due to the fact that these facilities purchase all their pharmaceuticals from the essential drug list." Whether there is a causal relationship needs to be confirmed. The message is reinforced by [32]: "Some suggestive findings include an association between increases in the supply of essential drugs (combined with training) and more appropriate use of medications in primary care settings."

Further research may be required to clarify how an essential drug prescribing environment improves prescribing behaviour. Perhaps part of the reason may be that knowledge of the relatively small list of essential drugs is more likely to reach a safe and effective level than knowledge of the much larger and constantly changing world of proprietary and non-essential drugs. A possible related factor is that knowledge of generic "essential drug" prescribing is less likely to be influenced and confused by pharmaceutical advertising.

An observational study of hospital management of common childhood illnesses in 21 hospitals in Bangladesh, Dominican Republic, Ethiopia, Indonesia, Philippines, Tanzania and Uganda found: "Inappropriate treatment with antibiotics, fluids, feeding, or oxygen, which accounted for 44% of all adverse factors, occurred in 80 (61%) patients. This category included both failure to give an indicated treatment and treatment given unnecessarily. Delay in giving appropriate treatment occurred in 24 (18%) patients, and inadequate monitoring, or failure to re-assess adequately during treatment, occurred in 39 (30%) patients" [25-27].

A study of knowledge and beliefs about epilepsy in Kenya showed that, at the community level, formally trained health workers may yet use traditional methods: "We poured paraffin on him. [The convulsions] stopped, and the eyes turned normal. Then [we] sent him to a mganga (witch doctor)" (Community health worker quoted in a focus group discussion) [10].

An observational study of health care providers (half of whom were nurses, and the rest clinical officers, doctors and pharmacists) looked at treatment of sexually transmitted diseases (STDs) in Nairobi, Kenya. It found that only 27% of the observed patients with STDs were managed correctly: "Quality of STD case management was unsatisfactory except in public STD-equipped clinics" [33].

#### Review papers and others

There were five informal reviews, two project descriptions, two intervention studies, one systematic review and one case-control study.

An informal review in *The Lancet*, and its accompanying comment from WHO staff, provide an overview that attempts to highlight the importance of meeting information needs, particularly at primary and district levels; the importance of local relevance and usability of information in addition to its reliability; and the advantages of "pull" approaches to meeting information needs, as compared with the prevalent model of industrialized countries' "pushing" information onto developing countries [4-6].

An earlier study, again in *The Lancet*, noted: "Much more research needs to be aimed at identifying information needs in less-developed countries. For example, what type of information is required: primary research, laboratory or clinical; reviews, systematic or non-systematic; print journals and books or on-line media; or information tools to support research, such as word processing or statistical packages?" [34-36]

Similarly, the *Report of the International Collaboration on Information Use in Cardiovascular Health Promotion in Developing Countries *pointed out that "before information technology can be used as an effective tool for promoting cardiovascular disease prevention, the information needs must be considered" [37].

There was one systematic review identified by our search, and this had the broad remit of "Strategies for integrating primary health services in middle- and low-income countries at the point of delivery". The review aimed to shed light on the ongoing debate between "vertical" and "integrated" approaches to primary health care, and concluded: "From the studies there was no clear evidence that integrating primary health care services improves the delivery of health services or people's health status in middle or low income countries" [37].

## Discussion

"The presence of CME [continuing medical education] materials in a hospital where I worked before coming here used to give me confidence and peace of mind about the management of surgical cases because I was able to perform certain procedures I had never performed before, just by referring to these materials and following the guidelines or instructions. Since I came here, I feel the gap ... I feel professionally 'insecure' without these materials ..." (Doctor) [12].

Given the small number of studies and the wide variation among the studies retrieved, their findings should be interpreted with caution. However, the studies do suggest a gross lack of knowledge about the basics on how to diagnose and manage common diseases [7,11,21,25-27,33], including CVDs and diabetes [17,18,20,24,28]. This lack of knowledge appears to go right across the health workforce and is sometimes associated with suboptimal, ineffective and dangerous health care practices. The implications are profound.

If this level of knowledge and practice is representative, as it appears to be, it indicates that modern medicine, even at a basic level, has largely failed the majority of the world's population. The human consequences are likely to be massive: death and harm caused directly by health workers; and failure to prevent deaths that are readily avoided by appropriate interventions. The numbers of people affected are likely to represent a daily catastrophe of huge proportions. The impact, however, is diffuse and often invisible, even unrecognized by health workers themselves.

Availability of health information provides confidence in clinical decision-making, improves practical skills and attitude to care [12,38] and alleviates professional isolation, yet this resource remains invisible in the complex health care systems. However, where information has been made available through manuals and treatment guidelines, reports show confusion and discrepancies between recommended care and practice [17,28,39], which underscores two points most often ignored – (1) improving the usability of materials (ensuring, for example, that guidelines are clear, easy to use, authoritative, referenced, in the right language, visually attractive and without unnecessary detail); and (2) training of health workers on the use of information. It is therefore emphasized that practical guidelines by experts in the field need to be developed with the active participation of end users, especially primary health care professionals [17] and there should be a better understanding of local needs. Thus, practical health care information should be user-driven, easy to use and accessible at the point of care.

### Information and learning needs

In this digital age, the growing urban-rural divide continues to influence the way health care professionals anywhere in the developing countries learn and gain access to information. For instance, in Africa in health facilities in capitals or medium-sized cities one can expect to have electricity and a telephone line, though both are rarely functioning 24 hours a day. In rural areas, however, such facilities are not always available and Internet connectivity is usually nonexistent [40]. This disparity in infrastructure limits the ability of health workers to take full advantage of information technologies in meeting their information and learning needs.

Understanding the local disease patterns is a major prerequisite to formulating appropriate strategies towards meeting the information needs of health care providers. In Africa, this pattern is changing rapidly. For instance, a recent review on the role of continuing medical education for health workers in Ghana noted that the country was being ravaged by both newly emerging infectious diseases such as HIV/AIDS and re-emerging infectious diseases such as malaria, TB and cholera. The authors cite local statistics that show that the incidence of noncommunicable diseases also continues to rise sharply, particularly in urban areas [8]. In the present literature review, over 50% of the publications focused on the major public health areas that cause significant morbidity and mortality in sub-Saharan Africa – (1) MCH; (2) HIV/AIDS, TB and STIs; and (3) CVDs and diabetes mellitus. Interestingly, the ratio of publications in these three broad categories was 3.0 : 1.5 : 1.0, respectively. It is imperative that resource allocation, including the provision of health information, takes into account the "priority diseases" [24] in any public health setting.

The rise in the prevalence of CVDs and diabetes mellitus in the developing countries presents new challenges to both patients and practitioners, particularly in Africa. There are clear deficiencies in the ability of health care workers to manage these patients [17,20,24,28]; many patients are underdiagnosed, while others die prematurely of preventable complications [24]. This is compounded by illiteracy and mistaken sociocultural beliefs among patients that militate against better management, especially for diabetes. In one study, the authors express concern that patients do not possess the necessary knowledge and understanding of the disease to use insulin safely [17]. One female patient is reported to have said: "There is no way that I can go on insulin because my husband will divorce me if I go on insulin".

Several studies illustrated the important role of health workers as health educators: one study [41] found that "The source of health educational interventions was more important than their frequency. Receiving health information through a physician or nurse was found to be a protective factor for diabetic complications as compared to mass media and health news." The quality of health education is clearly dependent on the knowledge of the health worker. A cross-sectional study of the quality of care provided to diabetic children in public children's hospitals in Egypt [42] showed "marked deficiencies in the provision of information to children with diabetes and their parents".

Health education is necessary for people to be aware of treatment possibilities. A study in rural Gambia showed that only 16% of people with active epilepsy were aware that it was possible to prevent attacks with medical treatment. The same study demonstrated how health education also needs to take into account social and cultural barriers and beliefs about health and illness: "the cause, persistence and treatment of epilepsy were accepted as ultimately under God's will and power" [7].

A number of studies suggested an association between availability of health care information (or lack of it) and knowledge or quality of health care:

• "We have shown that appropriate investigations are strongly associated with access to literature based scientific evidence (access to medical library, use of local journals and local and western textbooks)" [15].

• Quote from an East African surgeon [13]: "Laparoscopic surgery is fast developing and textbooks are not able to keep up with it. Before doing a complex laparoscopic surgery I read all the material on the subject available in the journals through this account and then make my plan. Frequently you will find descriptions and tips which are not in books."

• Quote from an East African surgeon [13]: "Four weeks ago I had to do penile reconstruction (after amputation for carcinoma). I searched abstracts and text to find about recent methods of reconstruction that is not involving microvascular surgery. I found several articles that helped me to make up my mind what to do."

• An article on the Blue Trunk Library (a WHO initiative that provides mini-libraries of books for district health care) noted "a discernible improvement in health care delivery services" [40] following the provision of the mini-libraries, but further details were not given.

This snapshot highlights the breadth and depth of the existing knowledge gaps among health practitioners that must be addressed in order to make health services safe for patients. These knowledge gaps contribute to the great fear among the general population of health services, and particularly hospitals, which are seen as places that will lead to your death rather than your cure. As one community health worker reported: "The father refused the mom to take the child to hospital because if he's injected and he has the charms he'll die". [10] This fear can extend to health workers themselves: in one of the papers reviewed, a community health worker declared: "I am in the faith which does not take children to hospital." [10]

### Methodologies for assessing information needs

"Information needs" is a complex, heterogeneous concept that encompasses several different perspectives, including:

• information needs or "wants" as perceived by health care providers

• information needs inferred by assessment of knowledge

• information needs inferred by assessment of health care practice.

Information needs or "wants" may include (1) recognition by health care providers of their own knowledge deficits and (2) identification by health workers of what they consider would be useful to improve their practice. Basing information provision on perceived needs is important, not least because it is more likely that information provided in response to expressed needs ("pulled" information) is more likely to be put into practice than information that is "pushed".

However, expressed needs are not the same as actual needs. Previous studies indicate a poor correlation between perceived knowledge deficiencies as reported by health care providers and actual knowledge deficits. And it is well known (although not described in the studies of this review) that the value of questionnaires of the type that ask "What books do you need?" is often limited by the respondents' knowledge of what is available: many have answered with titles of books they once used as students 30 years previously that are no longer in print.

Also, information needs of health workers are not fixed. Every individual health worker has unique information needs. Furthermore, a health worker's perceived and actual needs change with time, place and clinical caseload. Needs vary also according to availability of diagnostic, treatment and referral facilities. And they may also be influenced by social and cultural factors.

Similarly, methods for assessing information needs vary. In this review, the studies focused mainly on nurses, doctors and health assistants in rural or urban settings. In general, the studies we reviewed looked at needs in three dimensions: needs perceived by health workers, knowledge deficits and/or observation of health care practice.

Five studies were based on literature review [4-6,8,24,34-36,43]. Studies using structured questionnaires were applied in a significant number of the studies [14,15,17,18]. These were followed by a mixture of qualitative and quantitative methods that used structured and semistructured interviews, observations and focus group discussions [7,10,33,38,28]. One study used qualitative methods alone [12], while others were descriptive prospective and case control studies [31,41].

It is well recognized that availability of relevant, reliable information is a prerequisite for effective care, but is only one of a complex range of factors that determine effective care. Many of the studies in this review looked at the closely related area of training needs. One study [43] claims to provide a practical method to evaluate health care services for diabetes and other chronic disease management in resource-poor settings.

A comprehensive discussion on the merits of various methodologies, especially in the assessment of information needs, is not possible on the basis of these studies. However, some important observations are made:

1. Assessment of information needs requires a mix of quantitative and qualitative research. Quantitative methods were particularly useful to identify deficits in knowledge and practice, and it is noteworthy that multivariate analysis is often required to try to unpick the many confounding factors. Qualitative methods are particularly important in assessing information needs, and are particularly useful for assessing needs as perceived by health care providers. Studies using qualitative methods tend to take longer [12] and require expert knowledge in the analysis and interpretation of the results.

2. It is highly appropriate to assess local information needs within the broader context of local epidemiology and health care services. The RAPIA (Rapid Assessment Protocol for Insulin Access) approach [43] promises to be a practical approach that can be readily adapted to evaluate health care services for chronic disease management in resource-poor settings.

3. Explicit knowledge from research studies is unlikely to lead us to a "complete understanding" of information needs. Our understanding of changing needs can be strengthened by "continuous information needs assessment" – for example, by capturing tacit knowledge through email communities among specific health groups [37].

4. Assessments of information needs are prone to bias, as indicated in the discussion sections of some of the papers reviewed [21]:

• In questionnaires and interviews, health workers may give information that in reality they do not practise.

• Health workers may prepare themselves for the interview or questionnaire, giving information in line with official guidelines, not reflecting their true practices.

• There may be self-selection bias if non-participating doctors are different from the participants, e.g. those with less knowledge or who do not follow guidelines may be more likely to refuse interviews or ignore the questionnaire. Respondents to questionnaires may not be typical of the wider group that is being studied. The authors of the study of East African surgeons [13], for example, recognized that "participants are a self-selected group that includes opinion leaders, teachers, and researchers of the region. Thus it is possible that their valuation of Western literature is higher than that of other surgeons practicing in Africa".

• Another problem is that respondents may say what they think the researcher wants to hear, or they may tailor their response to benefit in some way: "It is not surprising that Ptolemy participants find Ptolemy the most useful gateway to the literature" [13].

• "Whenever observation methods are applied, the question arises of whether the presence of the observer may cause a Hawthorne effect, in the sense that the health care worker may have followed the treatment guidelines and the essential drug policy more rigorously than usual" [30].

5. Assessments of knowledge and practice produced two kinds of results: specific (e.g. "only 33 (66%) knew the most important symptoms of TB [and] only four doctors prescribed the correct regimen") and non-specific (e.g. 90% had "poor knowledge of the disease"). The specific results are easier to interpret and it would be easier to monitor changes in the future with specific knowledge gaps.

### Limitations of the review

This review used a single combination of search terms; other combinations and other search terms would inevitably yield a large number of additional papers, some of which would contain information relevant to the review. The authors of this review are aware of other relevant publications that were not identified by this search and which are therefore not included in this review.

The review is restricted to articles published in the last 10 years. It is possible that earlier articles may shed further light.

The reviewers were able to gain access to most, but not all, of the articles identified.

The selection of the 149 potentially relevant papers, from among the 1762 papers retrieved by the Medline search, was made on the basis of title and abstract alone; it is likely that some of the 1613 unselected articles may have contained valuable information.

Of the 149 articles retrieved, 35 were selected for detailed study. The remainder may have contained useful information. Furthermore, almost all of the 149 articles retrieved contain a list of references. These references were not explored, but are highly likely to yield further useful information for the review.

The original brief for this review included search of other databases such as WebSPIR, Biblioline, CABDirect, Web of Science and LISTA. However, due to resource constraints, it was agreed to restrict the review to Medline. This is an important limitation, as Medline indexes only a small proportion of the formal medical literature, and, in particular, excludes the vast majority of scholarly literature published in African journals. Also, Medline does not include the vast body of informal literature that might contain useful information (e.g. PhD and MSc theses, evaluation reports, proceedings of meetings and the content of email discussion lists).

## Recommendations

The review is to provide the first phase of a broader literature review of the information and learning needs of health care providers in developing countries, and ways of addressing those needs, for the benefit of the wider international development community.

### Extension of the current review

Given its limitations as described above, this review must be seen as a preliminary glimpse of the total literature on health care information needs. The review may be seen as a foundation on which to build in various ways:

• review of the full text of the remaining 114/149 papers retrieved by the current search strategy;

• use of alternative search strategies on Medline;

• search global databases other than Medline, e.g. WebSPIR, Biblioline, CABDirect, Web of Science, LISTA;

• search regional databases, e.g. African Index Medicus, African Journals OnLine.

If the review is to have real impact, it needs to (1) engage all stakeholders and (2) be seen as part of a wider process with a clear global objective.

1. Engaging stakeholders: The current review may be made freely available and presented as a starting point for us all to build on as a constantly evolving, living document to which others can add. Health care providers, publishers, librarians, researchers, development professionals and others may be invited to comment and discuss the key points emerging from this review. An open invitation could be posted to solicit new publications, reports and other material that may be considered for integration into the review. A mechanism for doing this is provided by two email groups that are specifically focused on the information and learning needs of health care providers: HIFA2015 and CHILD2015 .

2. Contributing to a clear global objective: Understanding the information and learning needs is recognized as fundamental to the global initiative, Healthcare Information For All by 2015 . The extension of the current review will (a) provide useful evidence and guidance for all those involved in the creation, exchange and use of health care information in the developing world; (b) identify gaps in our understanding of information needs, which will help researchers to identify priorities for future information and communication research; and (c) provide the evidence base that is needed to raise awareness worldwide, and particularly among international agencies and major donors, of the urgent need to strengthen political and financial commitment to meet the information and learning needs of health care providers in the developing world.

### Develop a tool for advocacy

The papers we have reviewed reveal a gross lack of knowledge about the basics on how to diagnose and manage common diseases. This lack of knowledge goes right across the health workforce and is associated with scandalously high levels of ineffective and dangerous health care. This global scandal needs to be communicated effectively to all those who could help make a difference. The authors suggest it would be useful to "map" this "knowledge gap" by collecting "killer facts" that illustrate lack of knowledge for a range of common diseases in different regions of the world. This could be a dramatically effective advocacy tool to promote the importance of meeting information and learning needs.

### Further work to document and describe processes and tools for assessing the information and learning needs of health care providers in developing countries

In order to do this, a multifaceted approach is probably needed, with guidance from experts in qualitative research. Such an approach would probably include database searching and interviews with experienced information researchers. It might also be valuable to organize an international seminar on the subject.

### Further information research

The current review suggests areas for future information research:

What information resources do health workers currently use?

None of the studies gave an overall picture of the level and type of information resources currently available to health workers. There is a need for further information on this, distinguishing between (1) reference materials at the point of patient care (i.e. on the health worker's desk, or carried by health workers during their daily work), and (2) learning materials that health workers may use to update knowledge.

Also, the studies did not shed much light on the relative availability and user preferences for different media (e.g. print, CD-ROM, Internet).

The review included one study that found that surgeons preferred "Western" to African literature – a finding that is opposite to previous studies of other cadres of health professionals. What kinds of "Western" and African literature are available to health workers in Africa; which do they prefer, and why?

#### Prescribing

"It was noted that all facilities had at least a drug formulary. Again there is need to compare data collected from facilities without such references to measure the impact on prescribing." [31] This kind of research would be simple to do, and it would also provide a baseline indicator of available information resources at each point of care (i.e. references within reach of the health worker at the point of care, e.g. on the table).

The study from Pakistan [28] found a high level of irrational and dangerous treatment of hypertension. Two thirds of the 1000 practitioners "relied on representatives from pharmaceutical companies for updates on information about antihypertensive medications". But the study did not look at whether there is an association or causal relation between dependence on pharmaceutical representatives and quality of practice. Provision of pharmaceutical marketing materials is widely perceived in the development community as a cause of wasteful and possibly harmful prescribing; hard evidence for or against this would be valuable for future interventions and advocacy. It would therefore be useful to assess the quality of health care practice among practitioners who depend on commercial pharmaceutical materials as compared with those who have access to generic formularies.

One of the studies from Tanzania [31] suggested a high quality of prescribing (for general patients as a whole) "due to the fact that these facilities purchase all their pharmaceuticals from the essential drug list." It would be useful to assess the relation between rational prescribing and the presence or otherwise of institutional approaches that focus on essential/generic drugs.

#### Patient safety

Only one author [12] noted the importance of critical incidents, and none of the studies looked at the interface between knowledge and patient safety. There appears to be a need for research to investigate the contribution of lack of knowledge or lack of information as a contributing factor to individual preventable deaths, especially where such deaths are caused by failures of health care.

#### Professional isolation

The comment from the Ugandan rural doctor ("Since I came here, I feel the gap. I feel professionally insecure without these materials") [12] suggests a real need to investigate further the link between lack of information and feelings of professional isolation and general dissatisfaction among rural health workers. How important is lack of information as a contributing factor to the rural-urban divide, the public sector-NGO divide and to the brain drain to other countries?

#### Language

The studies revealed little about the central issue of language. Given that most available health care information is in English, it would be interesting to compare, for example, an English-speaking African country, such as Ghana, with a French-speaking country in West Africa with a comparable level of economic development (e.g. Togo).

#### Guidelines

Five studies [16,17,25-29] found problems with understanding and/or implementation of international and/or national guidelines. The South Africa study, mentioned above, looked at national guidelines. Also a review on India and diabetes, stated: "There are no specific treatment guidelines proposed by the WHO for India or SE Asia. As a result, there are serious deficiencies in the standard of care that can be expected by patients when they consult their physician for diabetes treatment." How do practitioners interpret international guidelines, and national guidelines, where they exist, for diabetes and hypertension? What are the implications for producers of additional reference and learning materials for these two major diseases?

A study from Kenya (care of umbilical cord) [16] identified inadequate national guidelines as a cause of insufficient knowledge and practice, with national guidelines directly contradicting international guidelines. This suggests a need to specifically assess the availability, quality and perceived usefulness of national guidelines, and the extent to which these guidelines are actually put into practice.

## Conclusion

Information needs of health workers in developing countries are varied and are constantly under the influence of multiple factors – professional, institutional, cultural and infrastructural. Meeting these needs requires a clearer and better understanding of the complex interrelationships between these factors. Thus, no single method is ideal in evaluating health information needs. A snapshot of the published literature highlights progress, challenges and opportunities.

In particular, the availability of health information provides confidence in clinical decision-making, improves practical skills and attitudes to care. Serious and widespread deficiencies in the existing knowledge and practice of health practitioners is a reminder of the crucial importance of improving the availability of relevant, reliable health care information – and its potential to radically improve health care worldwide.

## Competing interests

The authors declare that they have no competing interests.

## Authors' contributions

NPW and FB jointly conceived, developed and reported this review.

## Supplementary Material

Additional file 1 **Publications included in literature review (references **[1-35]**).** Click here for file
