# Supplementary material for: Information needs of health care workers in developing countries: a literature review with a focus on Africa
Source: Hum Resour Health. 2009 Apr 8;7:30. doi: 10.1186/1478-4491-7-30 (PMC2680393; doi:10.1186/1478-4491-7-30)
Supplement: Additional file 1 — Publications included in literature review (references [1-35]). [file 1478-4491-7-30-S1.doc]

| **Ref** | **Method** | **Evaluation of:**  **1 = perceived needs**  **2 = knowledge deficits**  **3 = health care practice** | **Population** | **Country** | **Health area** |
| --- | --- | --- | --- | --- | --- |
| 1 | Description of project | N/A | N/A | N/A | General |
| 2 | Informal review | N/A | N/A | International | General |
| 3 | Interviews | 1, 2 | Village health workers and community members | Gambia | Epilepsy |
| 4 | Questionnaire | 1 | Whole range of health service personnel | Ghana | General |
| 5 | Questionnaire | 1 | Doctors in tertiary hospital | Kenya | Psychiatry |
| 6 | Interviews | 1, 2 | Doctors, patients, families | Kenya | Epilepsy |
| 7 | Survey | 1 | Doctors and patients in public-sector primary health care centres | South Africa | MCH: child nutrition |
| 8 | Interviews | 1 | Nurses, doctors and clinical officers | Uganda | General |
| 9 | Questionnaire | 1 | Surgeons and other hospital doctors | Kenya, Tanzania, Uganda | Surgery |
| 10 | Questionnaire | 1 | Doctors and other health workers | Indonesia | Care of elderly |
| 11 | Questionnaire | 2 | Doctors | China, Egypt, India, Kenya, Thailand | Adult pneumonia |
| 12 | Interviews | 2 | Mothers and primary health workers | Kenya | MCH: care of newborn |
| 13 | Survey | 1 | Doctors and patients in public-sector primary health care centres | South Africa | Diabetes |
| 14 | Questionnaire | 2 | Doctors and patients | India | Diabetes |
| 15 | Questionnaire | 2 | Consultants, junior doctors and nurses in a large teaching hospital | Saudi Arabia | Hypertension (BP measurement) |
| 16 | Interviews | 2 | Doctors and patients | Nigeria | Hypertension |
| 17 | Interviews | 2 | Doctors | Somalia | TB |
| 18 | Interviews | 2 | Trained TBAs | Gambia | MCH: postpartum haemorrhage |
| 19 | Questionnaire | 2 | Patients | Egypt | Diabetes |
| 20 | Informal review plus description of local experience | N/A | Various | International plus Kenya | Diabetes |
| 21 | Questionnaire and direct observation | 2, 3 | Doctors and other health workers in hospital facilities | Bangladesh, Dominican Republic, Ethiopia, Indonesia, Philippines, Tanzania, Uganda. | MCH: management of serious childhood illness |
| 22 | Questionnaire | 2 | GPs | Pakistan | Hypertension |
| 23 | Retrospective analysis of patient records, interviews, direct observation | 2, 3 | Doctors and other health workers in district hospitals | South Africa | MCH: child malnutrition |
| 24 | Direct observation | 3 | Nurse prescribers in nine primary health centres | Burkina Faso | General (prescribing habits) |
| 25 | Retrospective analysis of patient records | 3 | Doctors in church-owned primary health care facilities | Tanzania | General (prescribing habits) |
| 26 | Informal review | N/A | N/A | International | General (prescribing habits) |
| 27 | Interviews and direct observation | 3 | Nurses, doctors and clinical officers in various facilities | Kenya | STIs |
| 28 | Informal review | N/A | N/A | International | General |
| 29 | Informal review | N/A | N/A | International | CVD |
| 30 | Measure effect of educational intervention | N/A | Family support workers | Botswana, Lesotho, Malawi, Namibia, Zambia, Zimbabwe | MCH: child disability |
| 31 | Measure effect of educational intervention | N/A | Doctors in tertiary hospital | South Africa | Diabetes |
| 32 | Description of project | N/A | N/A | International | General |
| 33 | Case-control | N/A | Patients | Egypt | Diabetes |
| 34 | Questionnaire and observation | 2, 3 | Doctors and other health workers | Egypt | Diabetes |
| 35 | Assessment of health care facilities and services | 3 | N/A | Mozambique, Tanzania and Zambia | Diabetes |
